# Spondylodiscitis in Pediatric Age: A Retrospective Cohort Study

**DOI:** 10.1097/INF.0000000000003534

**Published:** 2022-06-07

**Authors:** Stefano Cavalieri, Benedetta Pessina, Giuseppe Indolfi, Luisa Galli, Sandra Trapani

**Affiliations:** From the * Postgraduate School of Pediatrics, University of Florence, Meyer Children’s Hospital, Florence; † Department of Pediatrics, San Jacopo Hospital, Pistoia; ‡ Department NEUROFARBA, University of Florence; § Pediatric Unit, Department of Health Sciences, Meyer Children's University Hospital, Florence, Italy; ¶ Infectious Diseases Unit, Meyer Children’s University Hospital; ‖ Department of Health Sciences, University of Florence, Florence, Italy

**Keywords:** spondylodiscitis, osteomyelitis, spine, infection, pediatrics

## Abstract

**Background:**

Pediatric spondylodiscitis is rare, hardly diagnosed and treated due to the nonspecificity of clinical presentation and laboratory investigations, difficulty of etiologic identification and lack of management guidelines.

**Methods:**

A retrospective study was conducted on 29 children with spondylodiscitis. Clinical, hematic and radiologic data were collected and compared between 2 age-subgroups (below and from 4 years old on) to investigate age-related differences. Epidemiologic, management and follow-up data were also described.

**Results:**

Slight male predominance and a peak of incidence <2 years were observed. Symptoms were significantly differently distributed in the 2 age-subgroups: children <4 years showed mainly refusal/inability to sit or bear weight, irritability, limping and poor general conditions; children ≥4 years most frequently had back pain and fever, and pain upon palpation of the spine. The lumbar spine and more than 1 vertebra were most frequently involved. Median diagnostic delay of 12 days was observed, without significant difference between age-subgroups, and delay >2 months was always associated with multivertebral involvement and complications. All children were treated with broad-spectrum antibiotics for a median of 12 weeks. Only in 1 complicated case, surgical treatment was also required.

**Conclusions:**

The clinical presentation of spondylodiscitis may be age-specific, with younger children often exhibiting subtle signs and symptoms. Broad-spectrum antibiotics covering for *Staphylococcus aureus* should be initiated as soon as possible and performed many weeks, being effective in treating the infection without clinical sequelae, even in patients with comorbidities. Surgical treatment should be reserved for complicated cases with neurologic involvement.

Spondylodiscitis describes a continuum of spinal infections, including discitis, vertebral osteomyelitis and spondylodiscitis itself, in which both the intervertebral disc and one or more vertebrae are involved.^1,2^ Spondylodiscitis is rare in children, with an incidence of 0.3 versus 2.4 per 100,000 in adults,^3,4^ representing only 3% of osteoarticular infections in pediatric hospitals.^5^ The first peak of incidence is observed in children 6 months–4 years of age, with a second peak in adolescents. Sporadic cases are reported in infants under 6 months of age.^6^ Spondylodiscitis is commonly acute (days/weeks) and caused by pyogenic bacteria, less often subacute/chronic (months/years) and mainly of tubercular origin.^7,8^ Patients usually present with back pain and refusal to bear weight or systemic symptoms, such as irritability and fever.^9–11^ Early diagnosis of pediatric spondylodiscitis is challenging, with diagnostic delays of up to 3 months due to its rarity and nonspecific onset.^12^ Blood tests are also nonspecific, with often normal white blood cell (WBC) count and slight increases in C-reactive protein (CRP) and/or erythrocyte sedimentation rate (ESR).^13^ Blood cultures show low sensitivity in most series.^6,12^ When etiological agents are isolated in culture, pyogenic bacteria are usually found, mostly *Staphylococcus aureus*, with methicillin-resistant *S. aureus* (MRSA) becoming more prevalent.^14^ Subacute/chronic spondylodiscitis is typically caused by nonpyogenic bacteria like *M. tuberculosis*, which is the most common cause of pediatric spondylodiscitis in developing countries.^15^ Conventional radiographs have low sensitivity, especially in the early stages of the disease. Magnetic resonance imaging (MRI) represents the standard for diagnosis, with high sensitivity and specificity.^16^ Bone biopsy can be performed when neoplasm cannot be excluded by imaging, antibiotic therapy is not effective or complications are present. Currently, there are no guidelines outlining the best therapeutic approach in childhood, while treatment guidelines for adults have been published.^17^ If not promptly diagnosed and adequately treated, spondylodiscitis can lead to severe orthopedic and neurological complications.^1^

In this retrospective study, we describe a series of pediatric spondylodiscitis cases. The aim of the study is to define the epidemiologic, clinical, radiological and microbiologic data of pediatric spondylodiscitis, and to search for age-related differences that could help in its diagnosis and management.

## METHODS

A retrospective study was conducted on children with spondylodiscitis hospitalized at Meyer Children’s University Hospital (Florence, Italy) from January 2010 to December 2021. Epidemiological and clinical findings, blood parameters, including WBC, ESR, CRP, microbiologic tests (either on blood or on bone biopsy by culture or real-time or broad-range polymerase chain reaction [*PCR*]assay or other), radiologic results, management, including type and duration of medical and surgical therapy were collected. Follow-up data including duration and outcome were also reported. The cohort has been divided into 2 age-subgroups (under and from 4 years old on) to investigate age-related differences, based on our sample age distribution and preexisting demographic data. Two cases of tuberculous spondylodiscitis were found and excluded due to the substantial differences with pyogenic spondylodiscitis. Statistical analyses were performed using IBM Statistical Package for Social Science software (SPSS, Version 28.0, Chicago, Illinois, USA). Data were reported as medians and interquartile ranges (IQR) for continuous variables and as percentages for categorical variables. The nonparametric Mann-Whitney *U* test was used for analyzing the differences in continuous variables between 2 groups of and the χ^2^ test was used for categorical variables. Values of *P* < 0.05 were considered statistically significant.

## RESULTS

Table 1 shows the details of the epidemiologic, clinical, hematologic and microbiologic characteristics of each patient.

**TABLE 1.** Summary of Epidemiological, Clinical, Hematologic and Microbiological Characteristics of All Patients (n= 29)

Cases	Sex/Age (years old)	Comorbidities, recent T/infection, animals	Symptoms	Fever	∆ (days)	WBC (x10^3^/mm^3^)	CRP(mg/dL)	ESR (mm/h)	Putative agent (identification technique)	Site of involvement	Complications
**1**	M/0.3	Neutropenia, bilateral BP, neuroenteric cyst	Irritability, refusal to bear weight, poor GC, feeding difficulty	Yes	81	17.6	14.3	–	MRSA (colture from purulent drained material)	D2-D6	Vertebral erosion, hump, E+PV abscess
**2**	M/1	Arnold-Chiari 1, GE	Irritability, refusal to bear weight	Yes	14	11.1	3	120	–	L5-S1	PV abscess
**3**	F/1.3	-	Limping, refusal to bear weight	No	14	9.1	0.48	11	*Salmonella spp.*(stool colture)	S1	No
**4**	M/1.3	–	Irritability, refusal to bear weight, back pain, axial hypotonia	No	13	9.4	1.83	25	–	D10-D11	No
**5**	F/1.4	Fever	Irritability, limping, refusal to bear weight, back pain	No	11	8.7	1.94	66	–	L4-L5	No
**6**	M/1.4	–	Irritability, limping, refusal to bear weight, Trendelemburg sign+	No	9	9.7	0.93	18	–	L3-L4	No
**7**	M/1.5	–	Irritability, limping, refusal to bear weight	No	10	15.9	1.79	61	–	L5-S1	E+PV abscess
**8**	M/1.7	–	Irritability, limping, refusal to bear weight, poor GC, constipation, opisthotonos	Low-grade	11	10.0	1.19	50	–	L3-L4	No
**9**	F/1.8	GE, cat	Irritability, limping, refusal to bear weight, poor GC, Lasegue sign+	No	8	17.9	0.42	47	–	L4-L5	No
**10**	M/1.8	Febrile seizures	Irritability, limping, refusal to bear weight, constipation, pain irradiated to legs	No	26	9.5	<0.29	9	–	L3-L4	No
**11**	M/1.8	minor T,GE	Irritability, limping, refusal to bear weight	No	6	11.4	1.57	18	–	L4-L5	No
**12**	F/2	Bike T	Irritability, limping, pain irradiated to legs	No	6	15.1	1.37	88	–	L5-S1	No
**13**	M/2.8	–	Back pain, limping, refusal to bear weight, poor GC	No	9	18.3	<0.29	27	*S. pneumoniae* (PCR on throat swab)	L3-L4	No
**14**	M/3.2	Polycystic kidney	Irritability, back pain, refusal to bear weight, poor GC, pain irradiated to legs	Low-grade	5	10.6	2.14	61	–	L5	PV abscess
**15**	M/3.2	–	Refusal to bear weight, pain irradiated to legs, night pain	No	12	7.7	0.34	72	–	L1-L2	E+PV abscess
**16**	F/4.3	Domestic T	Back pain, refusal to bear weight	No	14	8.3	<0.29	19	–	D10-D11	PV abscess
**17**	M/6.2	Arnold-Chiari 1, minor T, pharyngitis	Back pain, limping, refusal to bear weight, pain irradiated to legs, lower limbs hyporeflexia and weakness	Yes	17	6.0	2.32	42	–	S2	No
**18**	F/7.7	Minor T	Back pain, refusal to bear weight, pain irradiated to legs, Lasegue sign+	Yes	10	11.0	2.23	41	–	S1	No
**19**	F/9.3	Myelomeningocele, hydrocephalus, bedsores, UTI	Asymptomatic	No	6	8.2	0.69	–	*S. aureus, S. agalactiae* (PCR on skin lesion swab)	L5-S1	No
**20**	F/10.2	Cat	Back pain, weight loss, night pain, pain irradiated to legs	Low-grade	18	7.0	0.66	79	*B. henselae*(PCR on purulent drained material, serology)	L3	No
**21**	M/11.3	Stairs T	Back pain, weight loss, night pain	No	130	6.9	1.84	61	–	D11-D12	No
**22**	F/11.5	Minor T	Back pain, limping, night pain, abdominal pain	Yes	15	5.0	1.32	23	*S. aureus* (blood culture)	L3	No
**23**	M/12.1	–	Back pain	Yes	126	7.2	1.12	54	–	D12-L1	No
**24**	F/13.8	–	Back pain, limping, night pain	Yes	7	5.0	17.4	90	–	L5	PV abscess
**25**	M/14.1	Cat	Back pain	Yes	66	8.2	2.12	98	–	D7-D9	Vertebral collapse, hump
**26**	M/14.8	–	Back pain, refusal to bear weight	Yes	6	14.8	23.4	102	–	L4-L5	E+PV abscess *Spinal cord compression*
**27**	M/15.6	Dystonia, behavior disorder	Back pain	No	59	7.0	<0.29	15	–	D12-L1	No
**28**	M/16.2	Tetraparesis after a car accident	Back pain, night pain	Yes	15	8.6	9.88	70	–	L3-L4	PV abscess
**29**	M/16.8	Cat	Back pain	Yes	4	6.1	7.43	29	*S. aureus* (blood culture)	L2-L3	PV abscess

∆ indicates time between symptoms onset and diagnosis; BP,bronchopneumonia; CRP, C-reactive protein; D, dorsal; E abscess, epidural abscess; E+PV: epidural and paravertebral; ESR, erythrocyte sedimentation rate; F, female; GC, general conditions; GE, gastroenteritis; L, lumbar; M, male; MRSA, Methicillin-resistant Staphylococcus aureus; PV, paravertebral; S, sacral;T, trauma; UTI, urinary tract infection; WBC, white blood cells.: ;

### Epidemiologic data

**FIGURE 1.**
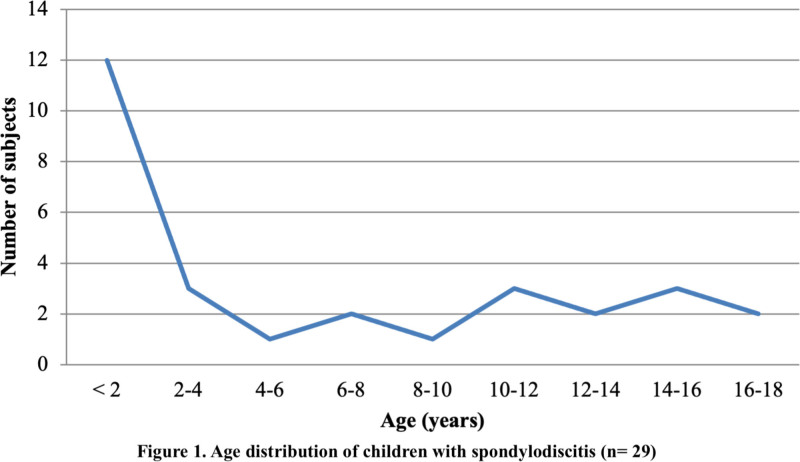
Age distribution of children with spondylodiscitis (n = 29).

Twenty-nine children, 19 (66%) males, with a median age of 3.2 years (IQR 1.7–11.5), were included; 15 (52%) were under the age of 4 years. A peak of incidence was observed in children <2 years (N = 12, 41%), with a subsequent homogenous distribution of cases with increasing age, and a slight increase in those who were over 10 years old (Fig. 1). Comorbidities were present in 7 (24%) patients: Arnold-Chiari type 1 malformation in 2 cases, immunosuppression in an infant with neutropenia, bronchopneumonia and neuroenteric cyst, and lumbosacral myelomeningocele with pressure sore, tetraparesis, polycystic kidney disease and dystonic movement-behavior disorder were present each in 1 patient. A previous trauma was referred in 7 (24%) patients and a recent infection was reported in another 24%; 4 patients had domestic cats.

Definitive diagnosis of spondylodiscitis was ascertained after a median of 12 days (IQR 8–17) from the clinical onset, with no significant difference in the 2 age subgroups. The diagnostic delay was greater than 2 months in 4 patients. Twenty-three patients (79%) had already been evaluated by a doctor for the same symptoms, before actual admission.

### Clinical Manifestations

The clinical presentation of all patients and divided by the 2 age subgroups is summarized in Table 2. The most common symptoms were back pain and refusal to sit and bear weight (N = 17, 59% and N = 18, 62%, respectively ). Fever was present in 11 (38%) patients, whereas 3 (10%) showed low-grade temperature; also limping (N = 13, 45%) and irritability (N = 12, 41%) were commonly found. Night pain was reported in 6 (21%) patients, worsening of general conditions in 5 (17%). Among the extra-skeletal manifestations, irradiation of pain to legs (N = 7, 24%) and abdomen (N = 1, 3%), weight loss (N = 2, 7%), new-onset constipation (N = 2, 7%) and feeding difficulty (N =1, 3%) were recorded. At physical examination, common signs were pain at spine palpation (N = 14, 48%), reduced hip mobility (N = 11, 38%), limited spine flexion (N = 9, 31%), paravertebral muscle contracture (N = 7, 24%), neck stiffness (N = 3, 10%) and hump (N = 2, 7%). Neurologic signs were present in 6 (21%) patients (#4, 6, 8, 9, 17, 18). Symptoms were differently distributed in the 2 age subgroups: children <4 years had mainly refusal/inability to sit or bear weight (N = 14, 93%), irritability (N = 12, 80%), limping (N = 10, 67%) and poor general conditions (N = 5, 33%); children ≥4 years most frequently showed back pain (N = 13, 93%) and fever (N = 9, 64%). A higher prevalence of pain upon palpation of the spine was referred in older patients (N = 9, 64% vs. N = 5, 33%), while neck stiffness was more recorded in younger subjects. No statistically significant differences regarding other clinical manifestations were found.

**TABLE 2.** Clinical Presentation of All Patients (n = 29), Divided by Age Subgroups

Number of patients (%)	Total 31 (n=31)	<4 years (n=17)	≥4 years (n=14)	*P* value
Temperature	29 (100)	15 (52)	14 (48)	
Fever (≥38.5°)	11 (38)	2 (13)	9 (64)	0.001*
Low-grade temperature (≥37.5°C)	3 (10)	2 (13)	1 (7)	0.154
Symptoms				
Back pain	17 (59)	4 (27)	13 (93)	<0.001*
Refusal/inability to bear weight	18 (62)	14 (93)	4 (29)	< 0.001*
Limping	13 (45)	10 (67)	3 (21)	0.014*
Irritability	12 (41)	12 (80)	0 (0)	<0.001*
Pain irradiated to the legs	7 (24)	4 (27)	3 (21)	0.742
Night pain	6 (21)	1 (7)	5 (36)	0.054
Worsening of general conditions	5 (17)	5 (33)	0 (0)	0.018*
New-onset constipation	2 (7)	2 (13)	0 (0)	0.157
Weight loss	2 (7)	0 (0)	2 (14)	0.129
Feeding difficulties	1 (3)	1 (7)	0 (0)	0.326
Abdominal pain	1 (3)	0 (0)	1 (7)	0.292
Physical findings				
Pain at palpation	14 (48)	5 (33)	9 (64)	0.096
Restricted hip mobility	11 (38)	7 (47)	4 (29)	0.316
Limited spine flexion	9 (31)	4 (27)	5 (36)	0.599
Paravertebral muscles contracture	7 (24)	3 (20)	4 (29)	0.590
Neurological signs	6 (21)	4 (27)	2 (14)	0.411
Neck stiffness	3 (10)	3 (20)	0 (0)	0.077
Hump	2 (7)	1 (7)	1 (7)	0.501

^*^: statistically significant

### Laboratory Workup

A normal WBC count on admission was present in 25 (86%) patients, with a median value of 9060/mm^3^ (IQR 7240–11,090). CRP was slightly elevated in most patients (N = 22, 76%), with median value of 1.57 mg/dL (IQR 0.66–2.23, normal value 0–0.5) and ESR was altered in most subjects (N = 21/27, 78%) with median value of 50 mm/h (IQR 24–71, normal value 0–20). No significant differences between age subgroups were observed in any hematological parameter. Five patients presented hypochromic microcytic anemia, and 4 were under 4 years old. Overall, microbiologic tests were positive in seven (24%) cases. Blood culture was collected in 12 (41%) patients; only 2 (17%) tested positive, both with isolation of Panton-Valentine Leukocidin-negative, methicillin-sensitive *S. aureus* (MSSA). *S. aureus* was isolated in the other 2 cases, 1 MRSA by culture on surgically drained material and 1 MSSA (together with *S. agalactiae*) by *PCR* on a pressure sore swab. *Bartonella henselae* was found on *PCR* on surgically drained material and on serology in 1 child, *Salmonella spp.* on stool culture in another subject, and *S. pneumoniae* on *PCR* on a pharyngeal swab in another. *PCR* on blood samples was performed in 22 (76%) patients, all with negative results. In 3 patients (10%) a biopsy of vertebral pathological tissue was undertaken, 1 of them without etiological identification, the other 2 identifying the previously described MRSA and *Bartonella spp*.

### Imaging Features and Complications

Radiological characteristics in our cohort are shown in Table 3. Spine radiography was the first radiological examination in 21 (72%) patients; 8 (38%) showed spinal alterations: in detail, physiologic lumbar lordosis was increased in 2 patients and decreased in other 2, and reduction of the intervertebral disc space was present in 4. Spine MRI was the diagnostic investigation in all cases. The median time between spine radiograph and MRI was 2 days (IQR 0–7). When the radiograph was altered, MRI was performed after a median latency of 1 day (IQR 0–3), whereas it was 5 days (IQR 0–15) if radiographs were normal. Spine CT, performed in 7 (24%) patients, was diagnostic in 6 (86%) cases. Technetium-99m bone scan was performed in only 1 patient and showed areas of increased tracer absorption.

**TABLE 3.** Radiological Characteristics of All Patients (n = 29), Divided by Age Subgroups

	Total	<4 years	Number of patients (%)≥4 years	*P*value
	29 (100)	15 (52)	14 (48)	
Site of involvement				
Thoracic	5 (17)	2 (13)	3 (21)	0.564
Thoraco-lumbar	2 (7)	0 (0)	2 (14)	0.129
Lumbar	14 (48)	9 (60)	5 (36)	0.340
Lumbo-sacral	5 (17)	3 (20)	2 (14)	0.411
Sacral	3 (10)	1 (7)	2 (14)	0.501
Disc involvement	25 (86)	15 (100)	10 (71)	0.026^*^
No of vertebrae involved				
1	8 (28)	2 (13)	6 (43)	0.075
2	19 (65)	12 (80)	7 (50)	0.089
≥3	2 (7)	1 (7)	1 (7)	0.960
Complications	11 (38)	5 (33)	6 (43)	0.597
Kyphosis and vertebral collapse	2 (7)	1 (7)	1 (7)	0.960
Spinal cord compression	1 (3)	0 (0)	1 (7)	n.s.
Epidural abscess	4 (14)	3 (20)	1 (7)	0.316
Paravertebral abscess	10 (35)	5 (33)	5 (36)	0.893
Psoas muscle displacement	2 (7)	2 (13)	0 (0)	0.142

^*^Statistically significant.

n.s. indicates not significant.

The lumbar spine was the most frequently involved site (N = 14, 48%), followed by the thoracic (N = 5, 17%) and lumbosacral (N = 5, 17%) tracts. The intervertebral disc was affected in 25 (86%), significantly more involved in younger children.

In 21 cases (72%) at least 2 vertebrae were affected. The 2 patients with 3 or more affected vertebrae had a diagnostic delay of more than 2 months and showed complications. Overall, 11 (38%) patients presented complications. The most frequent ones were paravertebral (N = 10, 34%) or epidural (N = 4, 14%) abscesses, with the secondary displacement of the psoas muscle by paravertebral abscess in 2 (7%) patients and secondary spinal cord compression by an epidural abscess in 1 (3%), detected on MRI. Kyphosis developed in 2 (7%) patients, always associated with vertebral collapse or destruction.

### Management

All patients received empirical intravenous (iv) antibiotics, mainly oxacillin (N = 21, 72%), ceftriaxone (N = 19, 66%), ceftazidime (N = 11, 38%) and teicoplanin (N = 10, 35%). The median duration of iv therapy was 4 weeks (IQR 3–5), with a subsequent switch to oral antibiotic therapy; the latter was performed for a median time of 8 weeks (IQR 5–9), with a median overall duration of antibiotic therapy of 12 weeks (IQR 9–12). The most used oral antibiotics were rifampicin (N = 17, 59%), amoxicillin-clavulanic acid (N = 15, 52%) and trimethoprim-sulfamethoxazole (N = 7, 24%). Surgical treatment was performed in 3 patients: a CT-guided percutaneous biopsy in 2 cases, and abscess drainage with biopsy in 1 child who underwent arthrodesis for vertebral stabilization 3 months later. Immobilization was indicated in 26 cases (90%), with a brace in 20 (69%), the remaining 3 patients, who had lumbosacral involvement, were told to refrain from sports. The median length of hospitalization was 26 days (IQR 19–34).

### Follow-up and Outcome

At discharge, only 7 (24%) patients reported residual symptoms, mainly mild back pain with mobilization and slight limping. One patient was lost at follow-up. All other patients underwent follow-up at the infectious disease outpatient unit for a median of 2 months (IQR 1–4.5) after the end of antibiotic therapy and 9 of them (32%) also had an orthopedic follow-up for a median of 19 months (IQR 14–29). Complete remission of symptoms occurred in all cases during follow-up. Radiologic follow-up was performed in 13 (46%) patients with spine MRI and radiography, in 14 (50%) patients with MRI alone, and in 1 (4%) with radiography alone. At the last radiologic follow-up, which occurred after a median of 12 months (IQR 8.5–29), complete normalization occurred in 4 (14%) patients; the remaining 24 (86%) showed minimal residual features during follow-up, which were not clinically relevant. None of our patients reported clinical sequelae at the end of follow-up nor experienced disease recurrence.

## DISCUSSION

Spondylodiscitis is a rare diagnosis in the pediatric age, undermining the lack of pediatric management guidelines and the frequent diagnostic delay. Our study represents one of the largest case series published in the literature, despite describing a relatively small cohort of 29 patients.

In accordance with other studies,^6,12^ in our cohort a slight male predominance was found. In larger series, a bimodal age distribution for this condition is reported,^6^ while in our study a high peak of incidence in young children (<2 years) was identified, with a median age at diagnosis of 3.2 years. By dividing our cohort into 2 age subgroups, distinct clinical pictures and pathophysiological mechanisms for each were observed: older children showed a typical clinical pattern characterized by higher fever and back pain, whereas in the younger ones, symptoms were subtle and mainly included irritability, refusal to bear weight, difficulty in changing position and deterioration of general conditions. This age-specific symptom distribution is widely reported in the literature.^6,9,18^ Due to clinical nonspecificity, misdiagnosis of spondylodiscitis is not uncommon; inflammatory arthritis, bone tumors and fractures are the main differential diagnoses.

Furthermore, routine blood tests are of little help in the diagnosis according to the literature.^10,19^ Also in our series, most patients showed normal WBC counts, whereas increased ESR was the most frequent inflammatory marker at diagnosis, and its normalization was observed before discharge in all patients but one, suggesting a possible correlation with antibiotic response.

Initial radiological examination with spine radiograph, performed in most patients, was negative in more than half. Suspicious radiograph findings led to earlier MRI performance, which was still required for diagnosis confirmation and complications detection. Suggestive features on MRI were reduced intervertebral disc height, disc hypointensity on T1-weighted images, disc hyperintensity on T2-weighted images or enhanced disc contrast. The lumbar spine was the most involved site, confirming previous pediatric data.^20^ The nonspecificity of symptoms and routine blood tests, along with frequent normal radiographs, contribute to the diagnostic delay, which was from about 2 weeks to up to 4 months, in our cohort. These data are in agreement with other series,^13,21,22^ however, Chandrasenan et al^13^ reported a greater diagnostic delay in children under 24 months, which was not observed in our cohort. A delayed diagnosis could imply more extensive bone infarction and consequent vertebral wedging/collapse, which can result in spinal instability and risk of spinal cord compression, especially in neonates and infants <6 months of age. Infection can spread to surrounding soft tissues causing paravertebral abscesses, or to the epidural and subdural space causing abscesses or meningitis.^2^ In our series, paravertebral and epidural abscesses were the most common complications. Two of the four patients with long diagnostic delay had three or more involved vertebrae and complications.

Pyogenic bacteria can reach the spine by the hematogenous route, by direct inoculation during a procedure or trauma, or by spreading from contiguous tissues. The most frequent mechanism in childhood is the hematogenous spread of a distant infectious focus, and a history of recent upper/lower respiratory tract infection or diarrhea is common. Many infants develop spondylodiscitis as a consequence of neonatal sepsis.^18,23^ In our cohort, comorbidities were reported in 24% of patients, and the patient with the most severe clinical picture was a neutropenic infant with previous pneumonia who developed severe complications. A history of trauma was present in 24% of our patients, similarly to other published reports.^24^ Recent infection was also variably reported in the previous series, in up to half of the cases according to Chandrasenan et al^13^ and Garron et al^21^, and was present in another 24% of our patients. Hematogenous spondylodiscitis usually develops starting from the vertebral body metaphyseal plate, which is highly vascularized in young patients; in older children, the intervertebral disc becomes progressively avascular and, therefore, bacterial infection directly locates in the vertebral body.^12,25,26^ Accordingly, in our cohort disc involvement was present in all children <4 years.

Etiological differences have become evident thanks to the availability of new diagnostic techniques. *K. kingae* has been recognized as the second leading cause of spondylodiscitis in children 6 months–4 years old, after *S. aureus*.^12^ However, in our series, this pathogen has not been identified. Blood cultures, collected in a few patients, were positive only in 2, both for *S. aureus*. According to the literature, the most frequently isolated pathogen was *S. aureus*, in 1 case MRSA; other isolates were *B. henselae, Salmonella* and *S. pneumoniae* (the last 2 with uncertain causative roles). Overall, microbiological investigations (by culture and *PCR* assays on different materials) allowed to identify the putative causative agent in 24% of patients, accordingly to other studies, where etiological identification rate ranges between 0% of Kayser et al^11^, Karabouta et al^27^ and Scheuerman et al^28^ series and 44% of Chandrasenan et al^13^ Garron et al^21^ and Kang et al^4^ reached higher identification rates (52% and 88%, respectively) due to the high number of biopsies performed in their cohorts (83% and 92%, respectively).^4,21^ In our series, biopsies were performed in 3 patients and were diagnostic in 2. Considering the good outcome of our patients after broad-spectrum antibiotic therapy, even in those without an etiological diagnosis, we confirm that the biopsy approach should be reserved for complicated and unresponsive cases requiring surgical treatment. All patients were treated with broad-spectrum antibiotic therapy administrated iv for a median of 4 weeks and then orally for an overall median duration of 12 weeks, similarly to what was reported in older studies.^29,30^ Other pediatric series reported a shorter duration of treatment, of 4–6 weeks overall.^4,20,27,28,31^ The most used iv agents in our cohort were oxacillin and third-generation cephalosporins, followed by teicoplanin. The most frequently administered oral antibiotics were semisynthetic penicillins and first-generation cephalosporins. In most of our cases, this management led to complete recovery without sequelae or relapses. Only 1 case has been also subjected to surgical treatment, with resolution. We, therefore, agree that empirical therapy should be based on iv broad-spectrum antibiotics covering for *S. aureus*, for at least 2 weeks, followed by several weeks of oral therapy; surgery should be reserved for cases with neurological signs of bone marrow compression.

The main limitations to this study are the retrospective design and the small sample size. Due to the data retrieval and collection method, some follow-up data were lost for patients hospitalized before 2018. Radiological follow-up was performed in all but 1 patient, with minimal residual findings in most of them. Clinical follow-up was performed in all patients at the infectious disease unit for about 2 months after antibiotic withdrawal; orthopedic follow-up was available for one-third of the patients. Despite the presence of comorbidities in approximately 24% of our patients, clinical remission was always obtained without clinical sequelae or relapse.

In conclusion, our study suggests that the clinical presentation of spondylodiscitis may be age-specific, with younger children often exhibiting subtle signs and symptoms, which should be taken into consideration in the diagnostic process. Treatment with broad-spectrum antibiotic therapy covering for *S. aureus* should be initiated as soon as possible; the duration of iv and oral antibiotic therapy should be tailored according to clinical and laboratory alterations. Patients with comorbidities and/or complications could require longer regimens. Surgical treatment should be reserved for complicated cases with neurological involvement. More studies are needed in pediatrics for the establishment of international diagnostic criteria and treatment recommendations.
